# Perinatal characteristics and pregnancy outcomes of advanced maternal age women with gestational diabetes mellitus: A retrospective cohort study

**DOI:** 10.1002/hsr2.1903

**Published:** 2024-02-25

**Authors:** Chen Jiang, Haiyan Wen, Tingting Hu, Yanfei Liu, Xiaoqing Dai, Yiming Chen

**Affiliations:** ^1^ Department of Medical Technology and Information Engineering Zhejiang Chinese Medical University Hangzhou Zhejiang China; ^2^ Department of Obstetrics Hangzhou Women's Hospital (Hangzhou Maternity and Child Health Care Hospital) Hangzhou Zhejiang China; ^3^ Department of Clinical Laboratory Hangzhou Women's Hospital (Hangzhou Maternity and Child Health Care Hospital) Hangzhou Zhejiang China; ^4^ Department of Prenatal Diagnosis and Screening Center Hangzhou Women's Hospital (Hangzhou Maternity and Child Health Care Hospital) Hangzhou Zhejiang China; ^5^ The Fourth School of Clinical Medical Zhejiang Chinese Medical University Hangzhou Zhejiang China

**Keywords:** advanced maternal age, adverse pregnancy outcome, chromosomal abnormality, gestation diabetes mellitus, retrospective cohort study

## Abstract

**Background and Aims:**

The prevalence of gestational diabetes mellitus (GDM) continues to increase, and the phenomenon of women giving birth at an older age is becoming more common worldwide. Less is known abouts the impact of GDM combined with advanced maternal age (AMA) on pregnancy outcomes. To explore the impact of AMA complicated with GDM on pregnancy outcomes.

**Methods:**

This study included 34,602 pregnancies between 2018 and 2020 in Hangzhou, China. The pregnant women were divided into four groups according to advanced age (≥35 years) and GDM as follows: AMA women without GDM (non‐AGDM) group (*n* = 2614), young pregnant women with GDM (YGDM) group (*n* = 4016), AMA women with GDM (AGDM) group (*n* = 850), and young pregnant women without GDM (non‐YGDM) group (*n* = 27,122). Univariate analysis was carried out by Mann–Whitney *U* test or Pearson's *χ* ^2^ test. Multivariate logistic regression analysis was used to investigate the effect of AMA and GDM on pregnancy outcomes.

**Results:**

Multivariate logistic regression analysis showed that in the comparison against non‐YGDM garoup, the ORs of fetal chromosome abnormality, parity, urgent cesarean section, gravidity, scheduled cesarean section, body mass index (BMI) ≥30 kg/m^2^, pre‐eclampsia, thrombocytopenia, hyperlipidemia, BMI 25–29.9 kg/m^2^, blood urea nitrogen, fasting blood glucose, and creatinine in AGDM group were 16.044, 4.284, 3.530, 3.284, 3.257, 2.049, 1.935, 1.898, 1.690, 1.471, 1.304, 1.216, and 1.026 (all *p* < 0.05).

**Conclusions:**

The prevalence of pregnant women with AGDM was 2.46% in Hang Zhou, China. The increasing gravidity of AMA women was related to a greater risk of GDM. The AGDM group associated with a greater risks of chromosomal abnormality in offspring and cesarean section, especially urgent cesarean section.

## 1 BACKGROUND

Gestational diabetes mellitus (GDM) is a frequent pregnancy‐related metabolic condition. GDM refers to any level of impaired glucose tolerance that appears or is initially detected during pregnancy.^1^ At present, pancreatic β‐cells fail to produce enough insulin to make up for a chronic fuel surplus, resulting in eventual insulin resistance, hyperglycemia, and an excessive supply of glucose to the developing fetus. Some evidence from other studies suggests that oxidative stress, placental factors, gluconeogenesis, low‐grade chronic inflammation, and adipose expandability are linked to the pathogenesis of GDM.^2^ In 2008, the hyperglycemia and adverse pregnancy outcome study found that maternal hyperglycemia independently increased the risk of adverse pregnancy outcomes, including premature delivery, cesarean section, larger than gestational age infants, need for intensive neonatal care, neonatal hypoglycemia, and neonatal hyperbilirubinemia.^3^ GDM associates with higher rates of adverse pregnancy outcomes such as macrosomia, primary caesarean section, intensive care unit admission, pre‐eclampsia, and clinical neonatal hypoglycemia.^4^ The prevalence of GDM varies in different regions; for example, GDM occurs in 5.50% of women in northern Greece,^5^ whereas it manifests in 5.20% of pregnant women in the United States.^6^ According to a meta‐analysis, mainland China had a 14.80% overall incidence of GDM (95% confidence interval [CI] 12.80%–16.70%).^7^ The incidence of GDM is steadily rising, and projections suggest that this upward trend will persist in the coming years,^8^ mostly due to the rising incidence of three primary risk factors, namely excessive weight gain during pregnancy, advanced maternal age (AMA), and obesity.^9^

AMA is defined as maternal age ≥35 years at delivery. AMA is associated with a wide range of adverse pregnancy outcomes, including chromosomal abnormalities, stillbirth, miscarriage, fetal growth restriction (FGR), pre‐eclampsia, GDM, preterm labor, and higher rates of caesarean section.^10^ AMA is associated with an elevated risk of GDM. A large research showed that women over 40 had a more than twofold higher risk of GDM than women under 30 in the United States (prevalence, 9.80% vs. 4.10%).^11^ When supposing the reasons why AMA women have worse pregnancy outcomes, there are multiple theories; for example, AMA increases the risk of placental vascular lesions, indicating AMA affects placental function.^12^ In a meta‐analysis, the incidence of adverse obstetrical and perinatal outcomes increased as AMA women aged.^13^ Furthermore, the biological and physiological changes related to advancing age associate with an increased likelihood of pre‐existing medical conditions, such as hypertension and diabetes, and a higher rate of obstetric complications, including antepartum hemorrhage and GDM.^14^ Yun and colleagues reported that age may not independently lead to adverse pregnancy outcomes,^15^ because older gravida have higher frequencies of associated risk factors such as hypertension and diabetes, and this may be the reason why AMA causes more adverse pregnancy outcomes. However, Li and colleagues showed that AMA women had more adverse outcomes compared to young women (non‐AMA) among a group of low‐risk pregnant women,^16^ indicating AMA was an independent strong risk factor. At present, there has been much research on the separate impact of GDM or AMA on pregnancy outcomes. Howerver, there is limited knowledge about the impact of AMA complicated with GDM on pregnancy outcomes. Deng and colleagues reported that GDM increased the risk of admission to the neonatal intensive care unit, neonatal‐assisted breathing, macrosomia, neonatal preterm delivery, neonatal poor Apgar score at 5 min, maternal admission to the intensive care unit, and mothers undergoing cesarean section among women with AMA.^17^ In another study, the risk of pre‐eclampsia was increased by advanced age and GDM.^18^

To investigate the effect of AMA and GDM (AGDM group) on adverse pregnancy outcomes and compare pregnancy outcomes of the AGDM group to the other three groups, namely, AMA women without GDM (non‐AGDM group), young pregnant women with GDM (YGDM group), and young pregnant women without GDM (non‐YGDM group) group, a retrospective cohort study was carried out to analyze 34,602 records from the Hangzhou Women's Hospital database. Of these, 850 women were in the AGDM group.

## 2 MATERIALS AND METHODS

### 2.1 Subjects

All data was obtained from a total of 34,602 pregnancies that were hospitalized at Hangzhou Women's Hospital from January 2018 to December 2020. We classified the 34,602 pregnancies into four categories as follows: AGDM group (*n* = 850), non‐AGDM group (*n* = 2614), YGDM group (*n* = 4016), and non‐YGDM group (*n* = 27,122). The study was restricted to singleton pregnancies achieved by natural conception. The Medical Ethics Committee at Hangzhou Women's Hospital granted approval for this study ([2019] Medical Ethics Review (4)−09).

### 2.2 Diagnostic and exclusion criteria

#### 2.2.1 Diagnostic criteria

AMA was defined as maternal age ≥35 years at delivery. Non‐AMA was defined as maternal age <35 years at delivery. GDM was defined as an oral glucose tolerance test of 75 g at 24–28 weeks of gestation according to the International Association of Diabetes and Pregnancy Study Group. Any one of the following criteria was used to diagnose GDM: fasting ≥92 mg/dL (5.10 mmol/L), 1 h ≥180 mg/dL (10.00 mmol/L), or 2 h ≥153 mg/dL (8.50 mmol/L).^19^

#### 2.2.2 Exclusion criteria

**Figure 1:**
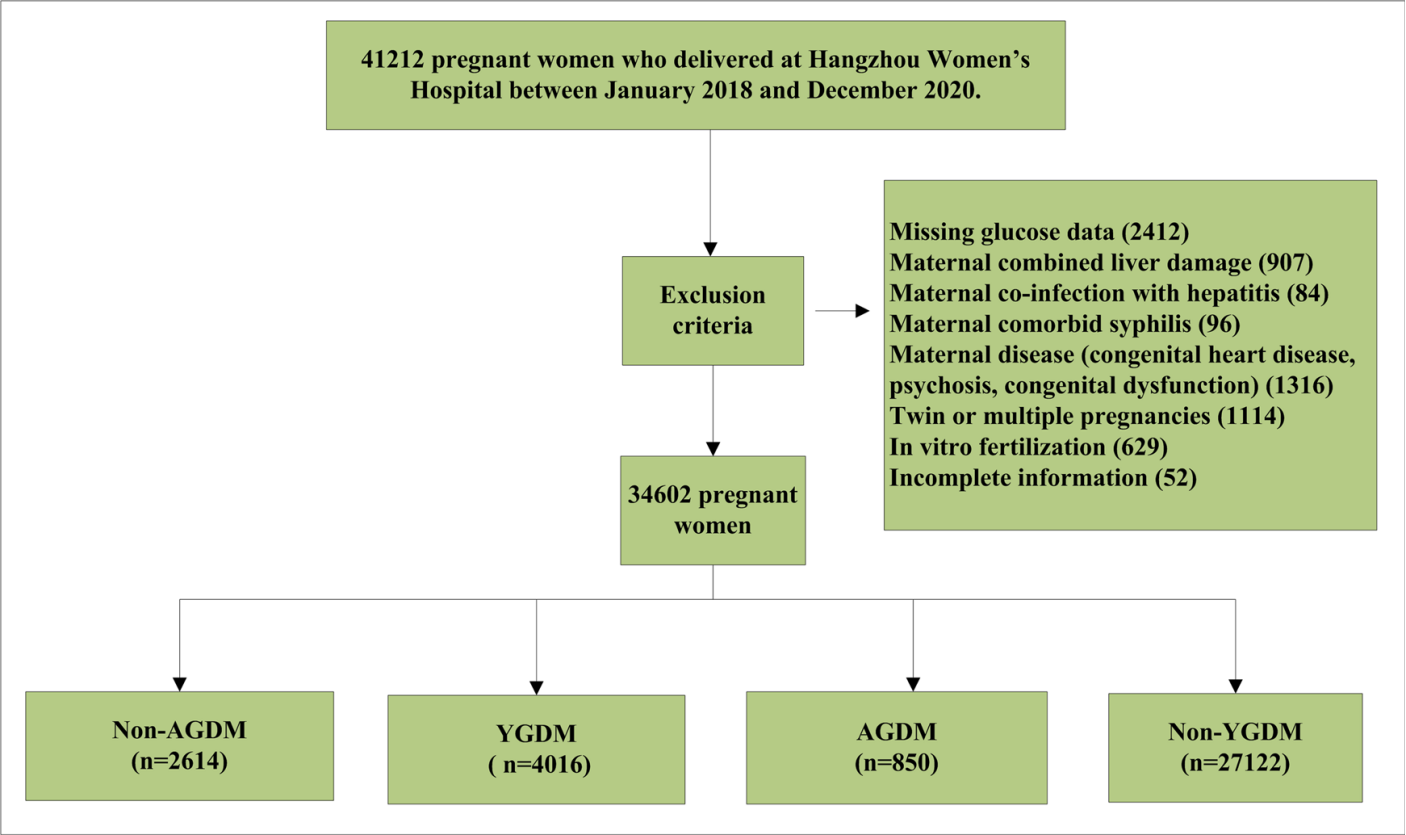
Flow chart of the selection of 34,602 pregnant women in this study. AGDM, advanced maternal age with gestational diabetes mellitus; non‐AGDM, advanced maternal age without gestational diabetes mellitus; non‐YGDM, young pregnant women without gestational diabetes mellitus; YGDM, young pregnant women with gestational diabetes mellitus.

Patients with a history of tumor resection, congenital dysfunction, mental disorder, liver damage, hepatitis, syphilis, and other infectious diseases before pregnancy were excluded. Women with twin or multiple pregnancies or who underwent in vitro fertilization (IVF) were excluded because of the intrinsic risk of multiple pregnancies and IVF. Patients with incomplete information were also excluded (Figure 1).

### 2.3 Blood urea nitrogen (BUN), creatinine (Cr), uric acid (UA), and fasting blood glucose (FBG) tests

#### 2.3.1 Detection reason and detection time

According to diabetes guidelines, hyperglycemia during pregnancy can cause temporary renal dysfunction in women with mild diabetic nephropathy, and pregnancy can cause permanent renal damage in women with severe renal insufficiency. BUN, Cr, and UA are sensitive indices of renal function, whereas FBG > 5.10 mmol/L is a diagnostic criterion of GDM. We assessed these parameters in the third trimester and on hospital admission.

#### 2.3.2 Instruments and reagents

The Beckman Coulter DXI‐800 full‐automatic biochemical analyzer (Beckman Coulter) was used for detection. BUN, Cr, UA, and FBG detection reagents were purchased from Beckman Coulter. All calibration reagents and quality control materials were used according to the manufacturer's instructions.

#### 2.3.3 Detection methods

The levels of BUN, Cr, UA, and FBG were measured in pregnant women that had fasted for 8–12 h. In brief, 3 mL of venous blood was collected in a vacuum tube containing separating gel, allowed to clot for 30 min, and centrifuged at 2000 rpm for 5 min. The levels of BUN, Cr, UA, and FGB were detected by the biochemical analyzer. Both internal‐quality and external‐quality controls were employed to guarantee the dependability of the test results in every test batch.

### 2.4 Definition of obstetric complications and pregnancy outcomes

All pregnancy complications and obstetric outcomes were collected from clinical data, and the diagnoses were made by obstetricians following Chinese guidelines.^20^, ^21^, ^22^ Hypertensive disorders of pregnancy (HDP) included gestational hypertension and pre‐eclampsia. Gestational hypertension was defined as blood pressure ≥140/90 mmHg. Pre‐eclampsia was defined as proteinuria and maternal blood pressure ≥140/90 mmHg. Proteinuria referred to protein in the urine ≥0.3 g/L in a 24‐h urine sample.^23^ The diagnosis of intrahepatic cholestasis of pregnancy (ICP) was established by the classic features of pruritus and elevated bile acids and liver enzymes. Hyperlipidemia was defined as a total serum triglyceride level ≥220 mg/dL (2.49 mmol/L) or a total serum cholesterol level ≥240 mg/dL (6.24 mmol/L). Antepartum hemorrhage was defined as vaginal bleeding after 24 weeks of gestation. Anemia was defined as a hemoglobin level <11 g/dL during pregnancy. Oligohydramnios referred to an amniotic fluid index (AFI) <5 cm, whereas polyhydramnios was characterized by an amniotic fluid index more than 25 cm or a vertical pocket measuring at least 8 cm. Fetal growth retardation (FGR) referred to an estimated weight on ultrasonographic examination below the 10th percentile adjusted to gestational age.^24^ Fetal distress referred to comprehensive life‐threatening symptoms such as abnormal fetal heart rate due to fetal hypoxia in the third trimester of pregnancy. Cord entanglement referred to the umbilical cord surrounding the fetal neck due to the umbilical cord being too long or the fetus being too small. Thrombocytopenia was defined as a platelet count of <150 × 10^9^/L. Placenta previa was defined as the placenta that completely or partially covers the inner cervix.^25^ Premature rupture of membranes (PROM) referred to the spontaneous rupture of the amniotic sac before the onset of labor. Placentae abruptio referred to the early separation of the placenta before childbirth.^26^ Obstetricians defined postpartum hemorrhage as an estimated blood loss ≥500 mL after vaginal delivery or ≥1000 mL after caesarean section. Chromosome abnormalities, either numeric or structural, included both mosaics, sex chromosome anomalies, and structural unbalanced karyotypes detected by microarray testing. Gestational age was divided into four groups as follows: early preterm (<238 days), late preterm (238–258 days), normal (259–287 days), and post‐term birth (>287 days). Cesarean section included urgent cesarean section and Scheduled cesarean section. Urgent cesarean section referred to one which required to deliver in time to reduce the risk to the mother or her newborn. Scheduled cesarean section was defined as one which was conducted before the onset of labor due to obstetric or medical indications or at the request of the pregnant woman. Apgar scores were defined as the average of neonatal Apgar scores at 1, 5, and 10 min. Low birth weight was defined as birth weight <2500 g, macrosomia was defined as >4000 g, and normal birth weight was defined as 2500–4000 g. body mass index (BMI) was divided into four groups as follows: <18.50 kg/m^2^, 18.50–24.99 kg/m^2^, 25–29.99 kg/m^2^, and ≥30 kg/m^2^.

### 2.5 Statistical analysis

Excel 2013 software was used for data collection. The statistical analysis was conducted utilizing the SPSS statistical program Version 21.0. Univariate analysis was carried out on continuous data or categorical data by Mann–Whitney *U* test or Pearson's *χ* ^2^ test. Multivariate logistic regression analysis was used to investigate the effect of AGDM on pregnancy outcomes by BMI, diastolic blood pressure, gravidity, parity, systolic blood pressure, mean artery pressure (MAP), BUN, Cr, UA, FBG, birth weight, birth length, HDP, thyroid function, hyperlipidaemia, antenatal anemia, thrombocytopenia, amniotic fluid volume, history of uterine scar, placenta previa, uterine inertia, antenatal hemorrhage, PROM, fetal distress, cord entanglement, mode of delivery, gestational age (days), fetal abnormality, dystocia, and postpartum hemorrhage. The results of multivariate logistic regression analysis were presented as adjusted odds ratios (ORs) along with 95% CI. A statistical significance level was established with a *p *< 0.05.

## 3 RESULTS

### 3.1 General characteristics of patients

A total of 34,602 singleton pregnancies were reviewed. Of these, 3464 (10.01%) were AMA and 31,138 (89.99%) were non‐AMA, whereas 4866 (14.06%) had GDM and 29,736 (85.94%) did not have GDM. The pregnant women were divided into four groups according to advanced age (≥35 years) and GDM as follows: non‐AGDM group (*n* = 2614), YGDM group (*n* = 4016), AGDM group (*n* = 850), and non‐YGDM group (*n* = 27,122). The proportions of these groups were 7.55%, 11.61%, 2.46%, and 78.38%, respectively. Maternal weight, maternal height, BMI, systolic blood pressure, diastolic blood pressure, MAP, gravidity, parity, BUN, Cr, UA, and FBG exhibited statistically significant differences (all *p* < 0.001) between the four groups, as shown in Table 1.

**Table 1:** General characteristics of the maternal groups.

Indicators	No. of cases *n* = 34,602	Groups				*Z/χ* ^2^	*p* Value
		Non‐AGDM (*n* = 2614)	YGDM (*n* = 4016)	AGDM (*n* = 850)	Non‐YGDM (*n* = 27122)		
Maternal weight (kg)	67.00 (62.00–73.00)	68.00 (63.00–74.00)	68.00 (62.00–75.00)	69.00 (63.00–76.00)	67.00 (62.00–72.00)	204.314	<0.001*
Height (cm)	160.00 (158.00–164.00)	160.00 (157.00–163.00)	160.00 (158.00–164.00)	160.00 (157.00–163.00)	160.00 (158.00–164.00)	75.724	<0.001*
BMI (kg/m^2^)	25.96 (24.17–27.99)	26.64 (24.89–28.62)	26.44 (24.39–28.69)	27.03 (25.10–29.24)	25.78 (24.03–27.73)	434.008	<0.001*
BMI (kg/m^2^)						409.834	<0.001*
<18.50	45 (0.1)	3 (0.1)	6 (0.1)	1 (0.1)	35 (0.1)		
18.5–24.99	12,251 (36.3)	680 (26.0)	1278 (31.8)	196 (23.1)	10397 (38.3)		
25–29.99	18,333 (53.0)	1572 (60.1)	2105 (52.4)	493 (58.0)	14163 (52.2)		
≥30	3673 (10.6)	359 (13.7)	627 (15.6)	160 (18.8)	2527 (9.3)		
Systolic blood pressure (mmHg)	118.00 (112.00–124.00)	117.00 (112.00–124.00)	119.00 (112.00–125.00)	119.00 (112.00–125.00)	118.00 (112.00–124.00)	27.074	<0.001*
Diastolic blood pressure (mmHg)	73.00 (68.00–79.00)	72.00 (67.00–78.0 0)	74.00 (68.00–81.00)	74.00 (68.00–81.00)	72.00 (68.00–79.00)	66.421	<0.001*
Mean artery pressure (MAP) (mmHg)	87.33 (83.33–93.33)	87 (82.33–93.00)	88.67 (83.67–94.67)	88.00 (83.00–95.00)	87.33 (83.00–93.00)	66.113	<0.001*
Gravidity						2291.252	<0.001*
≤1	17,102 (49.4)	307 (11.7)	1945 (48.4)	84 (9.9)	14766 (54.4)		
>1	17,500 (50.6)	2307 (88.3)	2071 (51.6)	766 (90.1)	12356 (45.6)		
Parity						3679.400	<0.001*
0	23,166 (66.9)	551 (21.1)	2761 (68.8)	180 (21.2)	19674 (72.5)		
≥1	11,436 (33.1)	2063 (78.9)	1255 (31.3)	670 (78.8)	7448 (27.5)		
BUN (mmol/L)	3.03 (2.50–3.67)	2.99 (2.42–3.60)	3.20 (2.60–3.89)	3.05 (2.40–3.80)	3.00 (2.50–3.61)	124.163	<0.001*
Cr (µmol/L)	58.00 (54.00–64.00)	59.00 (54.00–65.00)	58.00 (53.00–64.00)	59.00 (54.00–64.00)	58.00 (53.00–64.00)	19.311	<0.001*
UA (µmol/L)	328.00 (280.00–382.00)	316.00 (273.00–368.00)	333.00 (283.00–390.00)	326.00 (280.00–376.25)	328.00 (281.00–383.00)	74.400	<0.001*
FBG (mmol/L)	4.35 (3.82–5.17)	4.32 (3.83–5.20)	4.58 (3.97–5.51)	4.58 (4.01–5.51)	4.31 (3.80–5.11)	268.059	<0.001*

*Note*: Data are expressed as median (P25, P75) and number of patients and percentage (in parentheses) of the total group; *p* Values were calculated by *χ* ^2^ test or Mann–Whitney *U* test.

Abbreviations: AGDM, advanced maternal age with gestational diabetes mellitus; BMI, body mass index; BUN, blood Urea Nitrogen; Cr, creatinine; FBG, fasting blood glucose; MAP, mean artery pressure; Non‐AGDM, advanced maternal age without gestational diabetes mellitus; Non‐YGDM, young pregnant women without gestational diabetes mellitus; UA, uric acid; YGDM, young pregnant women with gestational diabetes mellitus.

* *p* < 0.001.

### 3.2 Distribution of perinatal traits across the maternal groups

The birth weight of the non‐YGDM group was lower than that of the non‐AGDM group, YGDM group, and AGDM group (3290 g vs. 3300 g, 3310 g and 3300 g, *p* < 0.001). There were statistically significant differences (*p* < 0.001) in birth length across the four groups. The Apgar scores and newborn gender did not exhibit any significant differences among the four groups (all *p* > 0.05), as indicated in Table 2.

**Table 2:** Distribution of perinatal traits across the maternal groups.

Indicators	No. of cases *n* = 34,602	Groups				*Z/χ* ^2^	*p* Value
		Non‐AGDM (*n* = 2614)	YGDM (*n* = 4016)	AGDM (*n* = 850)	Non‐YGDM (*n* = 27,122)		
Birth weight (g)	3300 (3030–3550)	3300 (3050–3550)	3310 (3050–3600)	3300 (3028–3603)	3290 (3030–3550)	31.143	<0.001*
Birth weight (g)						41.522	<0.001*
＜2500	1177 (3.4)	98 (3.7)	150 (3.7)	41 (4.8)	888 (3.3)		
＞4000	1410 (4.1)	101 (3.9)	217 (5.4)	52 (6.1)	1040 (3.8)		
2500–4000	32,015 (92.5)	2415 (92.4)	3649 (90.9)	757 (89.1)	25194 (92.9)		
Birth length (cm)	50 (50–50)	50 (50–50)	50 (50–50)	50 (50–50)	50 (50–50)	22.681	<0.001*
Apgar scores (points)	10 (10–10)	10 (10–10)	10 (10–10)	10 (10–10)	10 (10–10)	1.972	0.578
Newborn gender						0.276	0.964
Female	16,505 (47.7)	1251 (47.9)	1902 (47.4)	409 (48.1)	12943 (47.7)		
Male	16,097 (52.3)	1363 (52.1)	2114 (52.6)	441 (51.9)	14179 (52.3)		

*Note*: Apgar scores referred to the average of the scores at 1, 5, and 10 min; data are expressed as Median (P25, P75) and number of patients and percentage (in parentheses) of the total group; *p* Values were calculated by *χ* ^2^ test or Mann–Whitney *U* test.

Abbreviations: AGDM, advanced maternal age with gestational diabetes mellitus; Non‐AGDM, advanced maternal age without gestational diabetes mellitus; Non‐YGDM, young pregnant women without gestational diabetes mellitus; YGDM, young pregnant women with gestational diabetes mellitus.

* *p* < 0.001.

### 3.3 Univariate analysis of influencing factors

The analysis revealed that maternal height, systolic blood pressure, maternal weight, diastolic blood pressure, BMI, gravidity, parity, BUN, Cr, UA, FBG, MAP, birth weight, birth length, HDP, thyroid function, hyperlipidemia, antenatal anemia, thrombocytopenia, amniotic fluid volume, history of uterine scars, placenta previa, uterine inertia, antenatal hemorrhage, PROM, fetal distress, cord entanglement, mode of delivery, gestational age (days), fetal abnormality, dystocia, and postpartum hemorrhage were associated with AMA and GDM (all *p* < 0.05). However, Apgar scores, newborn gender, ICP, FGR, maternal arrhythmia, and placental abruption did not show any statistically significant differences among the four groups (all *p* > 0.05), as shown in Tables 1, 2, 3, 4.

**Table 3:** Distribution of pregnancy complications across the maternal groups (%).

Indicators	No. of cases *n* = 34,602	Groups				*χ* ^2^	*p* Value
		Non‐AGDM (*n* = 2614)	YGDM (*n* = 4016)	AGDM (*n* = 850)	Non‐YGDM (*n* = 27,122)		
Hypertensive disorders of pregnancy						100.712	<0.001*
Pre‐Eclampsia	558 (1.6)	49 (1.9)	110 (2.7)	21 (2.5)	378 (1.4)		
Gestational hypertension	1201 (3.5)	90 (3.4)	202 (5.0)	51 (6.0)	858 (3.2)		
Blood pressure normal	32843 (94.9)	2475 (94.7)	3704 (92.2)	778 (91.5)	25886 (95.4)		
Intrahepatic cholestasis of pregnancy						3.019	0.389
No	33960 (98.1)	2568 (98.2)	3928 (97.8)	833 (98.0)	26631 (98.2)		
Yes	642 (1.9)	46 (1.8)	88 (2.2）	17 (2.0)	491 (1.8)		
Thyroid function						13.989	0.030**
Hypothyroidism	2763 (8.0)	179 (6.8)	316 (7.9)	52 (6.1)	2216 (8.2)		
Hyperthyroidism	69 (0.2)	4 (0.2)	13 (0.3)	1 (0.1)	47 (0.2)		
Normal	31770 (91.8)	2431 (93.0)	3687 (91.8)	797 (93.8)	24855 (91.6)		
Hyperlipidaemia						77.494	<0.001*
No	33286 (96.2)	2494 (95.4)	3775 (94.0)	805 (94.7)	26212 (96.6)		
Yes	1316 (3.8)	120 (4.6)	241 (6.0)	45 (5.3)	910 (3.4)		
Maternal arhythmia						0.417	0.937
No	34475 (99.6)	2604 (99.6)	4000 (99.6)	846 (99.5)	27025 (99.6)		
Yes	127 (0.4)	10 (0.4)	16 (0.4)	4 (0.5)	97 (0.4)		
Antenatal anemia						30.111	<0.001*
No	26149 (75.6)	1945 (74.4)	3158 (78.6)	670 (78.8)	20376 (75.1)		
Yes	8453 (24.4)	669 (25.6)	858 (21.4)	180 (21.2)	6746 (24.9)		
Thrombocytopenia						11.705	0.008**
No	34164 (98.7)	2570 (98.3)	3962 (98.7)	831 (97.8)	26801 (98.8)		
Yes	438 (1.3)	44 (1.7)	54 (1.3)	19 (2.2)	321 (1.2)		
Amniotic fluid volume						50.753	<0.001*
Oligohydramnios	1931 (5.6)	109 (4.2)	172 (4.3)	34 (4.0)	1616 (6.0)		
Polyhydramnios	193 (0.6)	28 (1.1)	18 (0.4)	9 (1.1)	138 (0.5)		
Normal	32478 (93.9)	2477 (94.8)	3826 (95.3)	807 (94.9)	25368 (93.5)		
History of uterine scar						2735.516	<0.001*
No	29891 (86.4)	1527 (58.4)	3480 (86.7)	471 (55.4)	24413 (90.0)		
Yes	4711 (13.6)	1087 (41.6)	536 (13.3)	379 (44.6)	2709 (10.0)		
Placental abruption						1.762	0.623
No	34435 (99.5)	2602 (99.5)	3997 (99.5)	843 (99.2)	26993 (99.5)		
Yes	167 (0.5)	12 (0.5)	19 (0.5)	7 (0.8)	129 (0.5)		
Placenta previa						39.964	<0.001*
No	34332 (99.2)	2571 (98.4)	3987 (99.3)	835 (98.2)	26939 (99.3)		
Yes	270 (0.8)	43 (1.6)	29 (0.7)	15 (1.8)	183 (0.7)		
Uterine inertia						14.713	0.002**
No	33317 (96.3)	2532 (96.9)	3835 (95.5)	831 (97.8)	26119 (96.3)		
Yes	1285 (3.7)	82 (3.1)	181 (4.5)	19 (2.2)	1003 (3.7)		
Antenatal hemorrhage						10.462	0.015**
No	32250 (93.2)	2412 (92.3)	3714 (92.5)	804 (94.6)	25320 (93.4)		
Yes	2352 (6.8)	202 (7.7)	302 (7.5)	46 (5.4)	1802 (6.6)		

*Note*: Data are expressed as number of patients and percentage (in parentheses) of the total group.

Abbreviations: AGDM, advanced maternal age with gestational diabetes mellitus; Non‐AGDM, advanced maternal age without gestational diabetes mellitus; Non‐YGDM, young pregnant women without gestational diabetes mellitus; YGDM, young pregnant women with gestational diabetes mellitus.

* *p* < 0.001

** *p* < 0.05.

**Table 4:** Univariate analysis of pregnancy outcomes (%).

Pregnancy outcomes	No. of cases *n* = 34,602	Groups				*χ* ^2^	*p* Value
		Non‐AGDM (*n* = 2614)	YGDM (*n* = 4016)	AGDM (*n* = 850)	Non‐YGDM (*n* = 27,122)		
Premature rupture of menbranes						96.614	<0.001*
No	26865 (77.6)	2170 (83.0)	3175 (79.1)	730 (85.9)	20790 (76.7)		
Yes	7737 (22.4)	444 (17.0)	841 (20.9)	120 (14.1)	6332 (23.3)		
Fetal distress						88.135	<0.001*
No	31229 (90.3)	2463 (94.2)	3668 (91.3)	807 (94.9)	24291 (89.6)		
Yes	3373 (9.7)	151 (5.8)	8 (8.7)	43 (5.1)	2831 (10.4)		
Cord entanglement						8.573	0.036**
No	24104 (69.7)	1822 (69.7)	2849 (70.9)	620 (72.9)	18813 (69.4)		
Yes	10498 (30.3)	792 (30.3)	1167 (29.1)	230 (27.1)	8309 (30.6)		
Mode of delivery						1615.304	<0.001*
Vaginal delivery	23015 (66.5)	1137 (43.5）	2557 (63.7)	325 (38.2)	18996 (70.0)		
Urgent cesarean section	5502 (15.9)	415 (15.9)	717 (17.9)	170 (20.0)	4200 (15.5)		
Scheduled cesarean section	5867 (17.0)	1032 (39.5)	718 (17.9)	342 (40.2)	3775 (13.9)		
Others	218 (0.6)	30 (1.1)	24 (0.6)	13 (1.5)	151 (0.6)		
Gestational age (days)						134.333	<0.001*
Early preterm (＜238)	342 (1.0)	32 (1.2)	44 (1.1)	10 (1.2)	256 (0.9)		
Late preterm (238–258)	1272 (3.7)	137 (5.2)	201 (5.0)	51 (6.0)	883 (3.3)		
Post‐term birth (＞287)	399 (1.1)	12 (0.5)	7 (0.2)	0 (0.0)	380 (1.4)		
Normal (259–287)	32589 (94.2）	2432 (93.1)	3764 (93.7)	789 (92.8)	25603 (94.4)		
Fetal growth retardation						7.170	0.305
No	34220 (99.5)	2601 (99.5)	3997 (99.5)	840 (98.8)	26982 (99.5)		
Early fetal growth restriction	19 (0.1)	2 (0.1)	1 (0.0)	2 (0.2)	14 (0.1)		
Late fetal growth restriction	163 (0.5)	11 (0.4)	18 (0.4)	8 (0.9)	126 (0.5)		
Fetal abnormality						40.907	0.006**
Stillbirth	143 (0.4)	18 (0.7)	10 (0.2)	2 (0.2)	113 (0.4)		
Structural abnormality	114 (0.3)	7 (0.3)	16 (0.4)	4 (0.5)	87 (0.3)		
Single umbilical artery	87 (0.3)	12 (0.5)	12 (0.3)	3 (0.4)	60 (0.2)		
Other malformations	27 (0.1)	1 (0.0)	7 (0.2)	0 (0.0)	19 (0.1)		
Chromosome abnormality	18 (0.1)	4 (0.2)	4 (0.1)	3 (0.4)	7 (0.0)		
Cardiac malformation	30 (0.1)	1 (0.0)	6 (0.1)	0 (0.0)	23 (0.1)		
Renal malformation	15 (0.0)	1 (0.0)	1 (0.0)	0 (0.0)	13 (0.0)		
Normal	34168 (98.7)	2570 (98.3)	3960 (98.6)	838 (98.6)	26800 (98.8)		
Dystocia						57.873	<0.001*
Shoulder presentation	57 (0.2)	12 (0.5)	7 (0.2)	2 (0.2)	36 (0.1)		
Breech presentation	1046 (3.0)	85 (3.3)	141 (3.5)	34 (4.0)	786 (2.9)		
Occipitoposterior position	139 (0.4)	5 (0.2)	16 (0.4)	0 (0.0)	118 (0.4)		
Occipitotransverse position	93 (0.3)	5 (0.2)	5 (0.1)	0 (0.0)	83 (0.3)		
Cephalopelvic disproportion	822 (2.4)	43 (1.6)	124 (3.1)	16 (1.9)	639 (2.4)		
Cervical dystocia	93 (0.3)	5 (0.2)	11 (0.3)	1 (0.1)	76 (0.3)		
Head delivery	32352 (93.5)	2459 (94.1)	3712 (92.4)	797 (93.8)	25384 (93.6)		
Postpartum hemorrhage						12.653	0.005**
No	32682 (94.5)	2476 (94.7)	3764 (93.7)	822 (96.7)	25620 (94.5)		
Yes	1920 (5.5)	138 (5.3)	252 (6.3)	28 (3.3)	1502 (5.5)		

*Note*: Data are expressed as number of patients and percentage (in parentheses) of the total group.

Abbreviations: AGDM, advanced maternal age with gestational diabetes mellitus; Non‐AGDM, advanced maternal age without gestational diabetes mellitus; Non‐YGDM, young pregnant women without gestational diabetes mellitus; YGDM, young pregnant women with gestational diabetes mellitus.

* *p* < 0.001

** *p* < 0.05.

### 3.4 Multivariate logistic regression analysis

Multivariate logistic regression analysis revealed that the ORs of fetal chromosome abnormality, parity, urgent cesarean section, gravidity, scheduled cesarean section, BMI ≥ 30 kg/m^2^, pre‐eclampsia, thrombocytopenia, hyperlipidemia, BMI 25–29.99 kg/m^2^, BUN, FBG, and Cr in the AGDM group were 16.044, 4.284, 3.530, 3.284, 3.257, 2.049, 1.935, 1.898, 1.690, 1.471, 1.304, 1.216, and 1.026 (all *p* < 0.05), compared to the non‐YGDM group. These variables were risk factors for the AGDM group. By contrast, fetal distress and UA were protective factors for AGDM (OR = 0.641, 0.999, *p* < 0.05).

**Figure 2:**
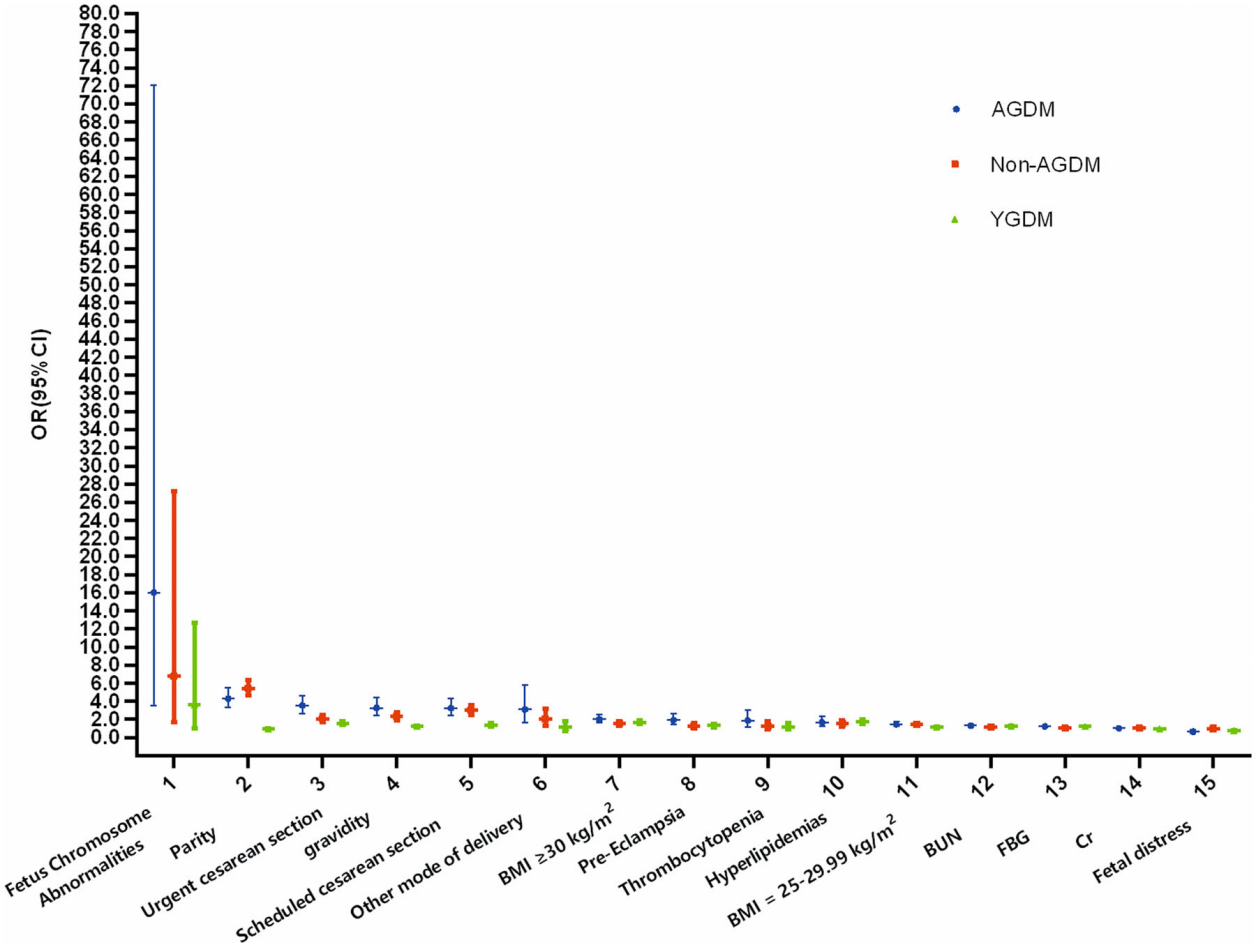
Odds ratios of related variables in the AGDM group, YGDM group, and non‐AGDM group by multivariate logistic regression analysis. AGDM, advanced maternal age with gestational diabetes mellitus; BMI, body mass index; BUN, blood urea nitrogen; Cr, creatinine; FBG, fasting blood glucose; non‐AGDM, advanced maternal age without gestational diabetes mellitus; Reference group was non‐YGDM, young pregnant women without gestational diabetes mellitus; YGDM, young pregnant women with gestational diabetes mellitus.

The ORs of fetal chromosome abnormality, early preterm (gestational age <238 days), hyperlipidemia, late preterm (gestational age 238–258 days), BMI ≥30 kg/m^2^, urgent cesarean section, scheduled cesarean section, pre‐eclampsia, BUN, FBG, gravidity, BMI 25–29.99 kg/m^2^, and UA in the YGDM group were 3.624, 1.876, 1.724, 1.721, 1.675, 1.591, 1.405, 1.304, 1.289, 1.232, 1.224, 1.139, and 1.001 (all *p* < 0.05). These variables were risk factors for YGDM. By contrast, post‐term birth (gestational age >287 days), occipitotransverse position, oligohydramnios, fetal distress, antepartum anemia, PROM, and Cr were protective factors for YGDM (OR = 0.088, 0.398, 0.653, 0.755, 0.844, 0.905, 0.988, all *p* < 0.05), see in Figure 2.

Fetal chromosome abnormality, parity, scheduled cesarean section, gravidity, urgent cesarean section, BMI ≥ 30 kg/m^2^, hyperlipidemia, antepartum hemorrhage, gestational hypertension, BMI 25–29.99 kg/m^2^, PROM, BUN, and Cr were risk factors for non‐AGDM (OR = 6.758, 5.442, 3.036, 2.326, 2.078, 1.568, 1.544, 1.540, 1.445, 1.427, 1.276, 1.175, and 1.038, all *p* < 0.05). UA was a protective factor for non‐AGDM (OR = 0.997, *p* < 0.05), as shown in Table 5.

**Table 5:** Multivariate logistic regression analysis of obstetric and perinatal outcomes.

Indicators/assignment	Groups								
	Non‐AGDM			YGDM			AGDM		
	OR	95% CI	*p* Value	OR	95% CI	*p* Value	OR	95% CI	*p* Value
BUN (mmol/L)	1.175	1.115–1.238	<0.001*	1.289	1.239–1.341	<0.001*	1.304	1.202–1.415	<0.001*
Cr (µmol/L)	1.038	1.032–1.044	<0.001*	0.988	0.983–0.993	<0.001*	1.026	1.016–1.036	<0.001*
UA (µmol/L)	0.997	0.997–0.998	<0.001*	1.001	1–1.001	0.001**	0.999	0.998–1.000	0.026**
FBG (mmol/L)	1.012	0.974–1.052	0.541	1.232	1.197–1.267	<0.001*	1.216	1.151–1.285	<0.001*
BMI (kg/m^2^)									
18.50	1.280	0.366–4.477	0.699	1.329	0.554–3.191	0.524	1.669	0.219–12.708	0.621
25–29.99	1.427	1.291–1.578	<0.001*	1.139	1.055–1.229	0.001**	1.471	1.236–1.749	<0.001*
≥30	1.568	1.350–1.822	<0.001*	1.675	1.496–1.875	<0.001*	2.049	1.625–2.583	<0.001*
18.50–24.99^a^									
Gravidity									
>1	2.326	1.960–2.760	<0.001*	1.224	1.119–1.338	<0.001*	3.284	2.445–4.412	<0.001*
≤1^a^									
Parity									
≥1	5.442	4.670–6.342	<0.001*	0.951	0.852–1.061	0.367	4.284	3.343–5.491	<0.001*
0^a^									
Gestational age (days)									
Early preterm (＜238)	1.176	0.653–2.117	0.590	1.876	1.180–2.982	0.008**	0.908	0.350–2.355	0.843
Late preterm (238–258)	1.243	0.988–1.563	0.063	1.721	1.431–2.069	<0.001*	1.283	0.896–1.836	0.174
Post‐term birth (＞287)	0.577	0.315–1.055	0.074	0.088	0.041–0.187	<0.001*	NA	NA	NA
Normal (259–287)									
Birth weight (g)									
2500	1.115	0.800–1.554	0.521	0.939	0.725–1.217	0.636	1.340	0.813–2.207	0.251
4000	0.802	0.636–1.011	0.062	1.179	1–1.391	0.050	1.290	0.934–1.782	0.122
2500–4000^a^									
Uterine scar									
Yes	0.936	0.785–1.115	0.458	1.006	0.857–1.180	0.944	0.927	0.704–1.220	0.589
No^a^									
HDP									
Pre‐Eclampsia	1.251	0.983–1.591	0.069	1.304	1.107–1.536	0.002**	1.935	1.415–2.648	<0.001*
Gestational hypertension	1.445	1.027–2.034	0.035**	1.258	0.996–1.588	0.054	1.184	0.712–1.970	0.516
Blood pressure normal^a^									
Hyperlipidaemia									
Yes	1.544	1.250–1.906	<0.001*	1.724	1.484–2.002	<0.001*	1.690	1.224–2.332	0.001**
No^a^									
Thyroid function									
Hypothyroidism	1.049	0.886–1.242	0.579	0.951	0.839–1.078	0.435	0.881	0.657–1.181	0.397
Hyperthyroidism	1.096	0.370–3.245	0.869	1.750	0.936–3.272	0.079	0.736	0.097–5.566	0.766
Normal^a^									
Amniotic fluid volume									
Oligohydramnios	0.921	0.743–1.143	0.455	0.653	0.553–0.772	<0.001*	0.753	0.523–1.085	0.128
Polyhydramnios	1.512	0.961–2.381	0.074	0.718	0.435–1.185	0.195	1.384	0.674–2.841	0.376
Normal^a^									
Antepartum anemia									
Yes	1.085	0.982–1.199	0.108	0.844	0.778–0.916	<0.001*	0.852	0.716–1.012	0.069
No^a^									
Uterine inertia									
Yes	0.942	0.657–1.351	0.746	1.244	0.957–1.617	0.102	1.237	0.598–2.559	0.566
No^a^									
Antepartum hemorrhage									
Yes	1.540	1.128–2.101	0.007**	0.969	0.706–1.329	0.843	1.096	0.649–1.851	0.731
No^a^									
Thrombocytopenia									
Yes	1.274	0.904–1.795	0.167	1.189	0.885–1.596	0.250	1.898	1.165–3.092	0.010**
No^a^									
Placenta previa									
Yes	1.060	0.714–1.572	0.774	0.772	0.508–1.175	0.228	1.321	0.727–2.399	0.361
No^a^									
Premature rupture of menbranes									
Yes	1.276	1.131–1.439	<0.001*	0.905	0.830–0.986	0.023	1.008	0.814–1.247	0.943
No^a^									
Fetal distress									
Yes	0.971	0.795–1.186	0.771	0.755	0.661–0.863	<0.001*	0.641	0.450–0.913	0.014**
No^a^									
Cord entanglement									
Yes	1.044	0.951–1.147	0.363	0.954	0.886–1.027	0.212	0.910	0.776–1.066	0.242
No^a^									
Fetal growth retardation									
Late fetal growth restriction	0.849	0.417–1.729	0.651	0.833	0.482–1.439	0.512	1.241	0.521–2.957	0.626
Early fetal growth restriction	1.121	0.205–6.143	0.895	0.313	0.039–2.517	0.275	3.215	0.515–20.065	0.211
Normal^a^									
Mode of delivery									
Urgent cesarean section	2.078	1.741–2.480	<0.001*	1.591	1.410–1.795	<0.001*	3.530	2.666–4.674	<0.001*
Scheduled cesarean section	3.036	2.541–3.628	<0.001*	1.405	1.227–1.609	<0.001*	3.257	2.451–4.327	<0.001*
Others	2.066	1.339–3.187	0.001**	1.146	0.730–1.799	0.555	3.110	1.662–5.819	<0.001*
Vaginal delivery^a^									
Dystocia									
Shoulder presentation	1.593	0.761–3.333	0.217	1.023	0.449–2.334	0.956	0.833	0.190–3.645	0.809
Breech presentation	0.956	0.738–1.237	0.730	1.018	0.830–1.248	0.863	1.029	0.699–1.514	0.885
Occipitoposterior position	0.902	0.356–2.285	0.828	0.865	0.506–1.479	0.596	NA	NA	NA
Occipitotransverse position	1.232	0.482–3.150	0.663	0.398	0.159–0.997	0.049**	NA	NA	NA
Cephalopelvic disproportion	1.079	0.762–1.528	0.670	1.061	0.851–1.322	0.600	0.853	0.492–1.478	0.570
Cervical dystocia	1.564	0.614–3.988	0.349	0.902	0.466–1.744	0.759	0.711	0.096–5.286	0.739
Head delivery^a^									
Postpartum hemorrhage									
Yes	0.996	0.659–1.504	0.985	1.086	0.741–1.591	0.672	0.729	0.335–1.583	0.424
No^a^									
Fetal abnormality									
Stillbirth	1.452	0.790–2.670	0.230	0.577	0.284–1.172	0.128	0.441	0.098–1.988	0.287
Structural abnormality	0.568	0.251–1.283	0.174	1.222	0.709–2.108	0.470	0.924	0.322–2.645	0.882
Single umbilical artery	1.934	0.968–3.864	0.062	1.359	0.725–2.547	0.339	1.629	0.491–5.400	0.425
Other malformations	0.649	0.083–5.047	0.680	2.149	0.876–5.269	0.095	NA	NA	NA
Chromosome abnormality	6.758	1.678–27.224	0.007**	3.624	1.037–12.666	0.044**	16.044	3.571–72.091	<0.001*
Cardiac malformation	0.435	0.056–3.383	0.426	1.433	0.570–3.606	0.445	NA	NA	NA
Renal malformation	1.139	0.129–10.067	0.907	0.554	0.072–4.283	0.572	NA	NA	NA
Normal^a^									

Abbreviations: AGDM, advanced maternal age with gestational diabetes mellitus; BMI, body mass index; BUN, blood urea nitrogen; Cr, creatinine; FBG, fasting blood glucose; HDP, hypertensive disorder complicating pregnancy; NA, beyond computation; Non‐AGDM, advanced maternal age without gestational diabetes mellitus; Reference group was non‐YGDM, young pregnant women with nongestational diabetes mellitus; UA, uric acid; YGDM, young pregnant women with gestational diabetes mellitus.

^a^ Reference.

* *p *< 0.001

** *p *< 0.05.

### 3.5 Comparison of influencing factors among groups

The risk of fetal chromosomal abnormality in the AGDM group was 16.04, 4.43 (16.044/3.624), and 2.37 (16.044/6.758) times higher than that in the non‐YGDM group, YGDM group, and non‐AGDM group, respectively. The risk of urgent cesarean section in the AGDM group was 3.53, 2.22 (3.530/1.591), and 1.70 (3.530/2.078) times higher than that in the non‐YGDM group, YGDM group, and non‐AGDM group, respectively. The risk of increasing gravidity in the AGDM group was 3.28, 2.68 (3.284/1.224), and 1.41 (3.284/2.326) times higher than that in the non‐YGDM group, YGDM group and non‐AGDM group, respectively.

## 4 DISCUSSION

We found that the prevalence of GDM was 14.06% and 10.01% in AMA parturients in Hangzhou, China (for the AGDM group, the proportion was 2.46%; for the YGDM group, the proportion was 11.61%). The increasing gravidity of AMA was related to an elevated incidence of GDM. Furthermore, AGDM increased the risks of urgent cesarean section and chromosomal abnormalities in offspring.

The incidence of GDM is influenced by factors such as race, ethnic background, mother age, and the diagnostic criteria used.^27^ In the present study, the prevalence of GDM was 14.06%, which was lower than the rate of 26.20% reported in public hospitals in Hadiya, southern Ethiopia^28^ and higher than the rate of 10.90% in Europe.^29^ However, our finding is consistent with the global prevalence of GDM in 2021.^30^ In the present study, the prevalence of AGDM was 2.46%, which was slightly lower than the prevalence of 2.73% reported by Lamminpaa et al.^18^

We found that the risk of fetal chromosomal abnormality in the AGDM group was 16.04, 4.43, and 2.37 times higher than that in the non‐YGDM group, YGDM group, and non‐AGDM group, respectively. The risk of fetal chromosomal abnormality increases significantly with AMA, especially for trisomy 21, trisomy 18, and trisomy 13. At 12 weeks of gestation, the risk of trisomy 21 increases from 1/1068 at maternal age 20 to 1/68 at maternal age 40, the risk of trisomy 18 increases from 1/2484 at maternal age 20 to 1/157 at maternal age 40, and the risk of trisomy 13 increases from 1/7826 at maternal age 20 to 1/495 at maternal age 40.^31^, ^32^ Compared to women aged 20–34, the aORs ranged from 2.09 to 2.74 for women aged 35–39 and from 5.95 to 8.53 for women aged >40 in a study that demonstrated the impact of AMA on chromosomal abnormalities.^33^ AMA is also associated with other complications such as spontaneous abortion, premature delivery, GDM, pre‐eclampsia, stillbirth, and increased cesarean section risk.^10^, ^34^ Chen and colleagues categorized pregnant women into three groups based on maternal age at delivery (<35 years, 35–39 years, and ≥40 years) and found that the rate of fetal malformations and fetal chromosomal abnormality rose as the maternal age increased.^35^ In another study, after controlling for variables such as first‐trimester exposures, mother age, education and BMI, a multivariate logistic regression analysis revealed that women with GDM had a 7.70‐fold increased likelihood (95% CI: 2.80, 21.10) of having a baby with a numeric sex chromosomal abnormality compared to those without GDM.^36^ GDM is also associated with increased oxidative stress, DNA damage, and chromosomal aberrations.^37^ Women of different ages with GDM have different pregnancy outcomes; for example, teens have a significantly increased incidence of PE and large‐for‐gestational‐age neonates, but a significantly decreased incidence of cesarean delivery. By contrast, there is a higher frequency of cesarean delivery and intra uterine fetal death, but a lower frequency of large‐for‐gestational age neonates in women aged 35–39 years.^38^

Our study showed that the rate of urgent cesarean section in the AGDM group (20.00%) was highest, compared to the non‐YGDM group (15.50%), non‐AGDM (15.90%), and YGDM group (17.90%), respectively. Multivariate logistic regression analysis showed that the risk of urgent cesarean section in the AGDM group was 3.53, 2.22, and 1.70 times higher than that in the non‐YGDM group, YGDM group, and non‐AGDM group, respectively, indicating AGDM increases the risk and rate of urgent cesarean section. Shames and colleagues reported that AGDM was associated with an higher rate of cesarean section,^39^ whereas Deng et al.^17^ demonstrated that AGDM women were at significantly increased risk of neonatal assisted ventilation (OR = 1.380), neonatal intensive care unit admission (OR = 1.436), neonatal low Apgar score at 5 min (OR = 1.034), neonatal high birth weight (OR = 1.132), premature birth (OR = 1.244), maternal intensive care unit admission (OR = 1.247), and cesarean section (OR = 1.193).

Our analysis also identified that the risk of increasing gravidity in the AGDM group was 3.28, 2.68, and 1.41 times higher than that in the non‐YGDM group, YGDM group, and non‐AGDM group, respectively. Luo and colleagues reported that among nulliparas, AMA was related to an increased likelihood of gestational hypertension (OR = 8.440), pre‐eclampsia/eclampsia (OR = 9.920), PROM (OR = 6.840), whereas among multiparas, AMA significantly increased the risk of GDM (OR = 3.290), anemia (OR = 1.850), polyhydramnios (OR = 3.290), PROM (OR = 5.140), and preterm labor (OR = 1.890).^40^ The recurrence rate of primiparas was lower than that of multiparas. (40.00% vs. 73.00%, *p* < 0.0001).^41^ Multiparity reduced the risk of preterm birth (adjusted risk ratio [aRR] = 0.910), low birth weight (aRR = 0.740), and small‐for‐gestational age (SGA) neonates (aRR = 0.670) compared to nulliparity. Thus, we should pay special attention to primiparas, especially those with AMA during antenatal care, for the purpose of reducing the risks of adverse birth outcomes.^42^

Table 1 indicated that the AGDM group had a higher BMI than the other groups, and there was a significant difference among the four groups (*p* < 0.001). BMI ≥ 30 kg/m^2^ and BMI 25–29.99 kg/m^2^ were high risk factors (OR = 2.049, 1.471) for the AGDM group, indicating increased BMI increases the incidence of GDM in AMA women. Dong and colleagues demonstrated that prepregnancy BMI was an independent risk factor for GDM, particularly in AMA women.^43^ Excessive weight gain in the first trimester is significantly associated with the incidence of GDM, regardless of the prepregnancy BMI. In women with GDM, pregestational BMI ≥ 25 kg/m^2^ and excessive gestational weight gain were significantly associated with increased infant birth weight.^44^ Gestational BMI gain was divided into three groups as follws: low (<4), medium (4–6), high (>6), the teams of gestational BMI gain <4 contributed to a higher prevalence of low birth weight and the teams of BMI gain >6 increased the risk of macrosomia.^45^ The YGDM group, non‐AGDM group, and AGDM group were associated with BMI 25–29.99 kg/m^2^, hyperlipidemia, BMI ≥ 30 kg/m^2^, BUN, UA, and Cr (all *p* < 0.05) compared to the non‐YGDM group, as shown in Table 5. There were no significant differences in the ORs of these variables in the three groups. Thus, the three groups had a similar risk effect or protective effect on BMI 25–29.99 kg/m^2^, hyperlipidemia, BMI ≥ 30 kg/m^2^, BUN, UA, and Cr.

Our study aimed to investigate the relationship between AMA or non‐AMA complicated with GDM status and pregnancy outcomes, which involved a relatively large sample size. However, our study still had several limitations. First, we excluded women with twin or multiple pregnancies and women underwent in vitro fertilization because these women had more clinical symptoms, poorer perinatal outcomes and a higher prevalence of GDM than women with single pregnancies conceived naturally. Second, while our study had a substantial sample size, its applicability is limited to Hangzhou, China. Future studies should include extended durations of observation and more extensive participant pools from diverse geographical areas. Third, our study lacked maternal education and lifestyle data, which may have an effect on pregnancy outcomes as well.

## 5 CONCLUSIONS

In conclusion, in Hangzhou, China, the prevalence of AGDM was 2.46%. The increasing gravidity of AMA women increased the risk of GDM. AGDM associated with a greater risk of chromosomal abnormalities in offspring and cesarean section, especially urgent cesarean section. Effective screening of chromosomal abnormalities should be performed in AGDM to avoid delivering an affected fetus. Enhancing personalized prenatal care is crucial in the management of AGDM to reduce the risk of adverse pregnancy outcomes.

## AUTHOR CONTRIBUTIONS

**Chen Jiang**: Data curation; formal analysis; writing—review & editing. **Haiyan Wen**: Resources. **Tingting Hu**: Writing—original draft. **Yanfei Liu**: Funding acquisition. **Xiaoqing Dai**: Writing—review & editing. **Yiming Chen**: Data curation; formal analysis; writing—original draft; writing—review & editing.

## CONFLICT OF INTEREST STATEMENT

The authors declare no conflict of interest.

## ETHICS STATEMENT

The study was undertaken with the endorsement of the Human Research Ethics Committee of Hangzhou Women's Hospital ([2019] Medical Ethics Review 2019‐4‐09). All patients provided informed consent to participate in the trial.

## TRANSPARENCY STATEMENT

The lead author Yiming Chen affirms that this manuscript is an honest, accurate, and transparent account of the study being reported; that no important aspects of the study have been omitted; and that any discrepancies from the study as planned (and, if relevant, registered) have been explained.

## Data Availability

The data sets used and/or analyzed in this study are obtained from the corresponding author according to reasonable requirements.
